# Feasibility, reliability, and validity of using accelerometers to measure physical activities of patients with stroke during inpatient rehabilitation

**DOI:** 10.1371/journal.pone.0209607

**Published:** 2018-12-31

**Authors:** Ji-Young Lee, SuYeon Kwon, Won-Seok Kim, Soo Jung Hahn, Jihong Park, Nam-Jong Paik

**Affiliations:** Department of Rehabilitation Medicine, Seoul National University College of Medicine, Seoul National University Bundang Hospital, Seongnam-si, Gyeonggi-do, South Korea; University of Maiduguri College of Medical Sciences, NIGERIA

## Abstract

Promoting physical activities is important for medical and functional recovery after stroke. Therefore, an accurate and convenient measurement of physical activities is necessary to provide feedback on functional status and effects of rehabilitative interventions. We assessed the feasibility, reliability, and validity of wearing accelerometers to monitor physical activities of stroke patients by estimating energy expenditure. This was a prospective observational quantitative study conducted in an inpatient rehabilitation unit. Twenty-four patients with subacute stroke were enrolled. They wore accelerometers on wrists and ankles for three consecutive weekdays. The feasibility was evaluated by daily wear-time. The test-retest reliability was determined by intra-class correlation coefficient. The validity was evaluated by comparing accelerometeric data to behavior mappings using Mann-Whitney U test, Spearman’s rho correlation coefficient (r) and Bland-Altman plots. Average wearing time for four accelerometers was 20.99 ± 3.28 hours per day. The 3-day accelerometer recording showed excellent test-retest reliability. For sedentary activities, wrist accelerometers showed higher correlation with direct observation than ankle accelerometers. For light to moderate activities, ankle accelerometers showed higher correlation with direct observation than wrist accelerometers. Overall, combined models of accelerometers showed higher correlation with direct observation than separate ones. Wearing accelerometers for 24 h may be useful for measuring physical activities in subjects with subacute stroke in an inpatient rehabilitation unit.

## Introduction

Physical activity (PA) is defined as “any bodily movements produced by skeletal muscles that result in energy expenditure (EE)” [1]. PA can decrease mortality and risks of diverse diseases [2]. Meta-analyses and systematic reviews have shown that increasing therapy and exercise time after stroke can result in better functional outcomes, including functional independence, walking ability, and the ability to perform activities of daily living [3]. Therefore, PA interventions should be emphasized for recovery in rehabilitation unit after urgent medical treatment for stroke. However, in reality, most stroke patients stay sedentary during free-living time in the rehabilitation unit at early stage [4, 5]. Besides, stroke survivors often get used to sedentary activity behaviors. They do not achieve adequate physical activities following discharge from rehabilitation [6, 7]. Therefore, activity-based rehabilitation program is necessary to increase PA in patients with stroke. Accurate measurement of both PA and PAEE (physical activity related energy expenditure) is also important to provide feedback on effects of PA interventions [8].

Many different methods are available to measure PA. However, there is no definite gold standard to measure PA in various clinical settings. Doubly labeled water method is considered the most accurate one. However, it has high-cost and its methodology is demanding [9, 10]. Various types of questionnaires, activity diaries, and functional tests have been developed to assess movements of upper and lower limbs. These questionnaires and functional tests such as Action Research Arm Test (ARAT) and Fugl-Myer Assessment scale (FMA) gather about patients’ ability to perform specific tasks, not their natural daily performances [11]. Although activity diaries can provide detailed information on certain physical activities, including activity types, patterns, purpose, intensity, duration, frequency, and body positions, the accuracy of these diaries is significantly affected by the participants’ cooperation [9]. These aforementioned tools, also known as indirect methods, are non-invasive, at low price, and convenient. However, they are vulnerable to a recall bias with over- and under- estimation [12]. Besides, patients with cognitive deficits cannot use these methods. Direct methods include direct observation and heart rate monitoring. Direct observations, also known as behavior mappings, allow monitoring of PA performance in various environments [13]. However, direct observation demands a large amount of mental and physical labor to record PA in detail. Devices to record heart rate might additionally increase the cost for purchase and maintenance [14].

Recently, motion detectors such as pedometers and accelerometers have been developed and marketed for research and commercial uses because they are portable and easy to wear. Accelerometers can provide continuous recording and quantification of PA and PAEE [15, 16]. Various accelerometers have proved intra- and inter- device reliability and validity [17]. However, few studies have reported the validity of accelerometers for stroke patients in rehabilitation hospital or home setting for the whole 24 h [18]. In addition, previous laboratory validations were conducted in “simulated free living” conditions where participants repeated certain physical performances [11, 18].

Therefore, this study was designed to evaluate the feasibility and reliability of accelerometers worn on extremities for 24 hours by monitoring PA in patients with subacute stroke during inpatient rehabilitation. The validity of accelerometers was further investigated by comparing its PAEE to behavior mappings. To improve the categorization of PA and the estimation of PAEE, accelerometers were worn on wrists and ankles, considering asymmetric functional uses of paretic limbs.

## Materials and methods

### Subjects

This was a prospective observational study conducted in an inpatient rehabilitation unit of Seoul National University Bundang Hospital from March 2015 to December 2015. Patients included in this study were 18 years or older with diagnosis of stroke. They were admitted to the rehabilitation unit after acute management. Exclusion criteria were: (1) traumatic brain injury; (2) symptoms attributable to other neurodegenerative disease such as dementia and Parkinson’s disease rather than stroke; (3) severe comorbidities such as infections and cardiopulmonary diseases; (4) impaired consciousness; (5) any skin problems in the area of accelerometer placement. The severity of functional impairment was assessed by National Institutes of Health Stroke Scale (NIHSS). Their disability and functional status were evaluated using modified Rankin Scale (mRS), Fugl-Myer Assessment scale (FMA), Korean version of Modified Barthel Index (MBI), and Functional Ambulation Category (FAC). Written informed consent was obtained from each subject. This study was approved by the Institutional Review Board of Seoul National University Bundang Hospital.

### Procedure

Accelerometeric recordings started on the first day of inpatient rehabilitation. Subjects participated in the rehabilitation every day, including at least one session of 30-minute physical therapy and one session of 30-minute occupational therapy. Subjects were instructed to wear accelerometers for three consecutive weekdays. The first day was used for acclimatizing to wearing accelerometers while the second and third days were used to obtain accurate and sufficient data. The 24-hour accelerometeric monitoring was repeated a total of three times. Subjects wore these accelerometers without cessation except when taking a shower. One accelerometer was placed on each limb. A wrist accelerometer was placed on the dorsal aspect of the wrist. An ankle accelerometer was placed just above the lateral malleolus. All accelerometers were fastened with Velcro bands.

Behavior mappings served as the standard criterion to validate accelerometeric data. Behavior mappings were conducted from 9 am to 5 pm each day for the 3-day accelerometeric recording period. Three observers conducted behavior mappings for each patient in 2–3 hour shifts. These observers were trained to record subjects’ PA every 10 minutes. The observation continued approximately 1 minute for every 10-minute time block. These observers recorded the location, contents of activity, and the highest intensity of the activity. Activities of different intensities were estimated as metabolic equivalent (MET) values based on Compendium of Physical Activities Tracking Guide expanded version. This enabled the estimation of the EE for the recorded PA (PAEE, kcal/min) by converting time spent in a specific PA to energy equivalents using the following equation:

PAEE(kcal/min)=METs×3.5×Bodyweight(kg)÷200

These MET values of activities were further categorized into four levels: sedentary (< 1.6 METs), light (1.6–2.9 METs), moderate (3.0–5.9 METs), and vigorous (≥ 6 METs). Results in terms of PAEE and MET values were compared to accelerometeric data.

Subjects and other medical staffs were informed that subjects’ activities were being monitored. Observers excused subjects’ private time such as using the rest room or changing clothes. Thus, accelerometeric data during private time were excluded from analyses.

### Equipment

ActiGraph wGT3X-BT (ActiGraph LLC, Pensacola, Florida, USA) was used. ActiGraph is a small (4.6 cm x 3.3 cm x 1.5 cm) and light (19 g) motion detector that provides information of bodily accelerations as 15-second epoch in three axes (vertical, Axis 1; anteroposterior, Axis 2; and mediolateral, Axis 3) at 30-100Hz with a dynamic range of ± 8 gravitation units. Activity counts (AC) from three sensing axes are integrated to vector magnitude (VM):

$$\text{VM}=\sqrt{{\left(\text{Axis}\;1\right)}^{2}{+(\text{Axis}\;2)}^{2}{+\;(\text{Axis}\;3)}^{2}}$$

Accelerometric data were stored directly into a flash memory. Subjects’ information including weight, height, age, and sex were manually entered into ActiGraph devices via ActiLife 6.8.2 (ActiGraph LLC, Pensacola, Florida, USA) software. ActiLife 6.8.2 calculates PAEE and MET from acceleration data using its proprietary algorithms [19]. A summary of wear time and non-wear time is also provided to exclude invalid data. Zero activity count lasting 60 minutes or longer was considered as non-wear period and not used for analysis. Freedson Combination ('98) and Work-Energy Theorem were used to convert vector magnitudes to PAEE:

Freedson Combination ('98) if Counts per minute > 1951 counts:

PAEE(kcal/min)=Scale×{0.00094×Countsperminute+(0.1346×Bodyweight(kg)−7.37418)}

Work-Energy Theorem if Counts per minute ≤ 1951 counts:

PAEE(kcal/min)=Countsperminute×0.0000191×Bodyweight(kg)

Freedson Adult (1998) was used to convert vector magnitudes to METs:

$$1\text{MET}=1\frac{\text{kcal}}{\text{kg}\times \text{h}}$$

Accelerometeric MET values were categorized into four levels in the same way as behavior mappings. Accelerometeric PAEE and MET values from 9 am to 5 pm for three monitoring days were then compared to behavior mappings.

### Data analysis

Descriptive analysis was performed for subjects’ characteristics and wearing time of accelerometers. Daily mean wearing time was used to determine wearing compliance. Test-retest reliability for the whole 24-hour AC data in terms of total vector magnitudes during 3 days was assessed using two-way random model intra-class correlation coefficient (ICC (2, 1)) with 95% confidence intervals.

PAEE and MET values from 9 am to 5 pm for three monitoring days were compared between accelerometers and behavior mappings. Various models of accelerometers were used to validate accelerometeric PA measurement, including the following: 1) affected upper limb (aUL), 2) affected lower limb (aLL), 3) unaffected upper limb (uUL), 4) unaffected lower limb (uLL), 5) affected upper and lower limb (aUL+aLL), 6) unaffected upper and lower limb (uUL+uLL), 7) bilateral upper limbs (bUL), 8) bilateral lower limbs (bLL), and 9) all limbs (bUL+bLL). The arithmetic mean value of PAEE was used for PAEE obtained from multiple accelerometers. For example,

aUL+aLL=(aUL+aLL)÷2,bUL+bLL=(aUL+aLL+uUL+uLL)÷4

Spearman’s rho correlation coefficient (r) was used to evaluate the relationship between EE obtained from accelerometers and EE calculated from behavior mappings. Differences in EE between accelerometers and behavior mappings were compared by Mann Whitney U test. Bland-Altman plots were used to examine the agreement of EE between accelerometer and behavior mappings. Limits of agreement were established as 1.96 SD from the mean difference [20]. All statistical analyses were conducted using software package SPSS, version 17.0 (SPSS Inc., Chicago, IL, USA). Correlation was considered weak when Spearman’s rho correlation coefficient (r) was between 0 and 0.25. The correlation was considered fair, moderate, or strong when r was between 0.25 and 0.5, between 0.5 and 0.75, or greater than 0.75, respectively [21]. Statistical significance was set at *p* < 0.05.

## Results

### Subject characteristics

Twenty-four patients were enrolled. Four subjects were excluded due to technical errors in initializing accelerometers, data downloading, or data storage (n = 1), non-wear time greater than 7.2 hours (10% of total monitoring time) (n = 2), and changed placements of accelerometers (n = 1). Consequently, the remaining 20 subjects who completed the monitoring were analyzed for this study. Demographic characteristics of these subjects are summarized in Table 1.

**Table 1:** Demographics and clinical characteristics of subjects.

Characteristics	Mean ± SD
Age (years)	59.7 ± 14.55
Time since stroke (days)	18.55 ± 8.34
Fugl-Myer Scale	
Upper extremity	27.40 ± 19.19
Lower extremity	20.75 ± 10.49
MBI	48.00 ± 23.74
	**n (%)**
Sex (male/female)	14/6 (70/30)
Stroke type (Ischemic/Hemorrhagic)	15/5 (75/25)
Side of hemiparesis (Right/Left)	8/12 (40/60)
NIHSS	
NIHSS≤ 7	13 (65)
NIHSS 8–16	7 (35)
NIHSS >16	0 (0)
mRS	
mRS 2	4 (20)
mRS 3	6 (30)
mRS 4	6 (30)
mRS 5	4 (20)
FAC	
FAC 5	2 (10)
FAC 4	3 (15)
FAC 3	4 (20)
FAC 2	4 (20)
FAC 1	4 (20)
FAC 0	3 (15)

MBI: Modified Barthel Index. NIHSS: National Institutes of Health Stroke Scale. mRS: modified Rankin Scale. FAC: Functional Ambulation Category

### Compliance

The average wearing time of accelerometers was 20.99 ± 3.28 hours per day. The average daily wearing time was 19.18 ± 4.32 hours for aUL, 19.77 ± 3.12 hours for aLL, 23.06 ± 1.33 hours for uUL, and 21.96 ± 1.83 hours for uLL.

### Test-retest reliability

Total vector magnitudes obtained from each day were analyzed for test-retest reliability of 24-hour accelerometeric monitoring. The test-retest reliability was high in 20 subjects during 3-day monitoring (Table 2). ICCs ranged from 0.953 to 0.980.

**Table 2:** Test-retest reliability of accelerometer data with 24-hour monitoring for 3 days.

	ICC	95% CI
Affected wrist	0.953	0.897, 0.981
Affected ankle	0.962	0.918, 0.985
Unaffected wrist	0.980	0.957, 0.992
Unaffected ankle	0.968	0.930, 0.987

ICC: Intra-class correlation coefficient; CI: Confidence interval.

### Validity

According to observers’ records, the average time spent was 352.5 ± 33.0 minutes for sedentary activities, 109.0 ± 47.5 minutes for light activities, and 18.0 ± 5.8 minutes for moderate activities. Vigorous physical activities were not detected.

The PAEE estimated from accelerometers during daytime activity (9 am to 5 pm) on weekdays were compared to those of behavior mappings. Total PAEE (kcal) was underestimated by all accelerometers. These accelerometers either under- or over-estimated PAEE (kcal/10min) according to activity intensities (Table 3). The PAEE of sedentary activities was overestimated by these accelerometers, particularly by wrist accelerometers (aUL, uUL, bUL), uUL+uLL, and bUL+bLL (*p<0*.*05*). In contrast, the PAEE of light and moderate activities was underestimated by these accelerometers, particularly by wrist accelerometers (aUL, uUL, bUL) and combinations of wrist and ankle accelerometers (aUL+aLL, uUL+uLL, bUL+bLL, *p<0*.*05*).

**Table 3:** Energy expenditure measured by behavior mappings and by accelerometers.

	Map	aUL	aLL	uUL	uLL	aUL+aLL	uUL+uLL	bUL	bLL	bUL+bLL
**Total PAEE (kcal)**	676.0(112.4)	613.8(106.6)†	611.8(123.7)†	650.2(144.0)*	657.2(165.6)*	612.0(108.0)†	653.7(145.2)*	632.0(112.2)†	634.5(140.7)†	633.2(121.7)†
**PAEE (kcal/10min)**										
Sedentary	10.5(2.5)	12.0(2.7)*	11.4(2.7)	13.2(4.0)†	11.7(3.8)	11.7(2.4)	12.5(3.4)*	12.6(2.9)*	11.6(3.1)	12.1(2.7) *
Light	22.0(2.7)	15.2(5.2)†	16.0(6.7)	15.0(5.0)†	18.2(6.1)	15.6(5.9)†	16.6(6.1)†	15.1(4.8)†	17.1(6.5)	16.1(6.2)†
Moderate	31.4(3.5)	15.2(6.0)†	29.6(7.4)	16.1(6.2)†	32.0(6.5)	22.4(6.7)†	24.0(7.1)†	15.6(5.8)†	30.8(7.3)	23.2(6.1)†
Vigorous	-	-	-	-	-	-	-	-	-	-

*, significantly different from estimated EE based on behavior mappings at 0.05 level (two-tailed).

^†^, significantly different from estimated EE based on behavior mappings at 0.01 level (two-tailed).

PAEE: Physical Activity related Energy expenditure; Map: Behavior mappings; aUL: affected Upper Limb; aLL: affected Lower Limb; uUL: unaffected Upper Limb; uLL: unaffected Lower Limb; aUL+aLL: affected Upper and Lower Limb; uUL+uLL: unaffected Upper and Lower Limb; bUL: bilateral Upper Limbs; bLL: bilateral Lower Limbs; bUL+bLL: All limbs.

Correlations of PAEE between accelerometers and behavior mappings were generally favorable (Table 4). For total PAEE (kcal), ankle accelerometers (aLL, uLL, bLL), bUL+bLL, and uUL+uLL showed strong correlations with behavior mappings. Wrist accelerometers (aUL, uUL, and bUL) and aUL+aLL showed moderate correlations. For PAEE (kcal/10min) of sedentary activities, bUL showed strong correlation with behavior mappings. For PAEE of light and moderate activities, bLL, bUL+bLL, uLL, and uUL+uLL showed strong correlations with behavior mappings.

**Table 4:** Correlations of energy expenditure between behavior mappings and accelerometers.

	Map- aUL	Map- aLL	Map- uUL	Map- uLL	Map- aUL+aLL	Map- uUL+uLL	Map- bUL	Map- bLL	Map- bUL+bLL
**Total PAEE (kcal)**	0.621*	0.784^†^	0.723^†^	0.790*	0.742^†^	0.841^†^	0.734^†^	0.867^†^	0.887^†^
**PAEE (kcal/10min)**									
Sedentary	0.669*	0.552*	0.704^†^	0.604*	0.674*	0.698^†^	0.784^†^	0.614*	0.747^†^
Light	0.643*	0.732^†^	0.688*	0.755^†^	0.702^†^	0.731^†^	0.727^†^	0.814^†^	0.759^†^
Moderate	0.598*	0.709^†^	0.632*	0.748^†^	0.723^†^	0.754^†^	0.621*	0.778^†^	0.753^†^
Vigorous	-	-	-	-	-	-	-	-	-

*, Correlation is significant at 0.01 level (two-tailed).

^**†**^, Correlation is significant at 0.001 level (two-tailed).

PAEE: Physical Activity related Energy expenditure; Map: Behavior mappings; aUL: affected Upper Limb; aLL: affected Lower Limb; uUL: unaffected Upper Limb; uLL: unaffected Lower Limb; aUL+aLL: affected Upper and Lower Limb; uUL+uLL: unaffected Upper and Lower Limb; bUL: bilateral Upper Limbs; bLL: bilateral Lower Limbs; bUL+bLL: All limbs.

**Fig 1:**
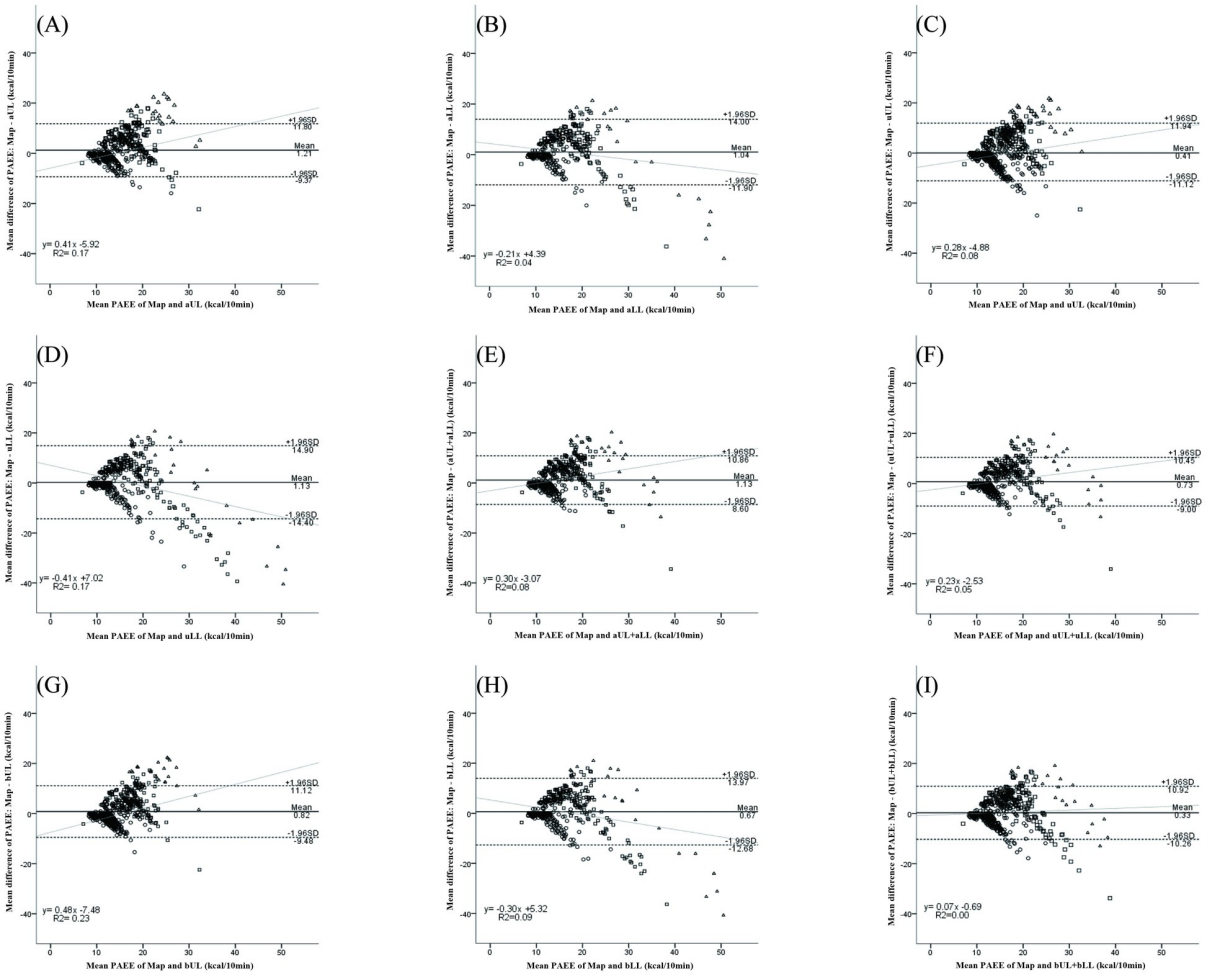
Bland-Altman plots showing energy expenditure agreements between accelerometers and behavior mappings. Filled circles (•) show behavior mappings. Open circle (○), triangle (Δ), and square (□) represent sedentary (< 1.6 METs), light (1.6–2.9 METs), and moderate (3.0–5.9 METs) activities from acccelerometers. Vigorous (≥ 6 METs) activity was not observed. Behavior mapping showed the strongest agreement with energy expenditure (PAEE) of bUL+bLL without proportional bias (R^2^ = 0.003, *p* = 0.052, Fig 1I). A relatively better agreement was observed in lower intensities than that in higher intensities. Dotted lines represent mean ± 1.96 SD of difference in PAEEs. Lightface lines represent regression line for difference in PAEEs.

Bland-Altman plots were used to analyze the agreement of PAEE between behavior mappings and accelerometers (Fig 1). Behavior mapping and bUL+bLL showed the strongest agreement without proportional bias (R^2^ = 0.003, *p* = 0.052, Fig 1I). A relatively better agreement was observed in lower intensities than that in higher intensities.

## Discussion

This study has demonstrated the feasibility, reliability, and validity of using diverse combinations of wrist and ankle accelerometers to monitor PA during early inpatient rehabilitation in stroke patients with various disability levels. Wearing accelerometers was bearable and PA measurement was reliably repeated for three monitoring days. The PAEE measured by accelerometers in different combinations showed good correlations with that one measured by behavior mappings.

The current study proved the feasibility of using accelerometers to monitor PA with an average wearing time of 20.99 ± 3.28 hours per day. Moreover, only a couple of subjects were excluded due to non-wear time (n = 2) or displacements of accelerometers (n = 1). Hence, accelerometers are realistic and unobtrusive tools to monitor everyday PA regardless of disabilities or comorbidities. Mansfield et al. have reported that more than 80% of participants with subacute stroke show compliance with wearing an accelerometer for 6 weeks during self-directed physical activity after discharge from rehabilitation [6]. Lang CE et al. have suggested that bilateral wrist accelerometeric monitoring over 24 hours is a practical and objective method to determine the amount of use of affected and unaffected limbs [22]. Prajapati et al. have also suggested that ankle accelerometers can be used in unobtrusive monitoring to evaluate characteristics and qualities of poststroke ambulation [23].

We placed accelerometers on wrists and ankles instead of hip or waist because they were seen practical and realistic in the clinical setting. Wrist and ankle accelerometers were easy to wear. In addition, they would cause little discomfort or displacement during rehabilitation. Furthermore, previous studies have shown that wrist and ankle accelerometers are as valid as hip and waist accelerometers. Melanson et al. have reported that wrist, hip, and ankle accelerometeric counts were significantly correlated with energy expenditure (kcal/min) during walking and jogging regardless of the location [24]. In addition, a wrist accelerometer was the best to predict energy expenditure for walking and jogging, accounting for 86% of the variance in energy expenditure [24]. Moreover, Montoye et al. have reported that ankle accelerometers were the most accurate ones among wrist, hip, and ankle accelerometers for predicting energy expenditure in both structured and simulated free-living settings [25].

In the current study, we shortened the conventional 7-day monitoring into 3 days to encourage participation and completion [26]. The 3-day accelerometeric recording showed sufficiently high test-retest reliability for measuring daily PA, consistent with previous studies on the number of days for the reliable data acquisition to evaluate PA. These studies have examined the adequate monitoring time to measure PA in healthy adults in a free-living environment. Scheers et al. have recommended monitoring during weekends with at least 3 weekdays to estimate habitual activities and 3 days to capture light activities [27]. Dillon et al. have proposed 3, 2, and 6 days to monitor sedentary, light, and moderate/vigorous activities, respectively [28]. Haeuber et al. have suggested that two separate 48-hour recordings may produce reliable data of community- based activities in chronic stroke [29].

Most patients with stroke are inactive. They stay in bed during hospital care. Their inactive time ranges from 24.2% to 98% (median: 48.1%) during the day [4]. Patients in our study remained sedentary for 352.5 ± 33.0 minutes, accounting for 73.3% of 480-minute daytime observation in behavior mappings. Since adequate physical activities bring aerobic effects that can promote neuronal plasticity and prevent deconditioning [30–33], it is important to reduce the sedentary time during inpatient rehabilitation using strategic approaches. Mansfield et al. have reported that rehabilitative intervention that provides group exercise and self-directed physical activity during inpatient period can lead participants to have higher motivations for recovery, fewer barriers to physical activity, and higher participation in physical activities after discharge [6].

In the present study, total PAEE (kcal) was underestimated by all accelerometers (Table 3). As accelerometers were set to detect bodily movements at 30-100Hz with 15-second epoch, the underestimation could be related to the low frequency, short duration, or low intensity of physical activities. Since higher frequencies do not directly result from voluntary muscle contraction, an impact of the PA (e.g., an impact between foot and walking surface) is necessary. Physical activities below 20Hz, too quick or swift, at a constant speed without gravitational acceleration [15], or low intensity (e.g., METs < 1.6) would not be detected by accelerometers well. PA of low intensities mainly in horizontal directions could not be detected by accelerometers well because accelerometers are more sensitive to acceleration in vertical directions [34].

An accelerometer is a convenient tool to quantitatively monitor and feedback PA in a stroke rehabilitation unit. In sedentary activities, wrist accelerometers correlated strongly with behavior mappings (Table 4, Fig 1). In addition, bUL estimated PAEE more accurately than unilateral monitoring whereas uUL significantly overestimated PAEE (Table 3, Fig 1). As movements from upper limbs are often quick and less effortful, wrist accelerometers, particularly on the unaffected side, may overestimate PAEE during sedentary activities. By contrast, ankle accelerometers estimated PAEE of sedentary movements precisely by comparison with the wrist ones. Movements of lower limbs required larger muscles and greater effort while many sedentary activities mainly involved movements of the upper body without motion of lower extremities.

To evaluate the PAEE of activities at light to moderate intensities, ankle accelerometers correlated with behavior mappings stronger than wrist accelerometers (Table 4). The discrepancy in PAEE measurement between affected and unaffected side was less prominent in lower limbs than that in upper limbs (Table 3). Patients with stroke had reduced arm sway due to hemiparesis or use of walking devices during ambulation. Consequently, the inevitable reduction of multi-axial movements from upper limbs exaggerated the underestimation or less accurate measurement of PAEE by wrist accelerometers compared to ankle ones. The lower limbs, on the other hand, had less prominent difference between affected and unaffected sides. As accelerometers are sensitive in bodily movement of vertical direction, lower limbs that mainly undergo vertical movements during physical activities (e.g., transfer, standing, and walking) [34] are subjected to accelerometeric detection regardless of paresis. This also implies earlier motor recovery of lower limbs than upper limbs after stroke.

In conclusion, our results suggest that wrist accelerometers are useful for evaluating sedentary activities. bUL is the most reliable and accurate while uUL is the most realistic and economical. Ankle accelerometers are useful for evaluating activities of higher intensities such as standing, transfer, and walking. bLL is the most reliable and accurate while uLL is the most realistic and economical. bUL and bLL help to measure PAEE accurately and to evaluate the discrepancy in PAEE between affected and unaffected side. uUL and uLL may be used when patients have difficulties in wearing accelerometers on bilateral wrists or ankles due to discomfort or medical problems. For an unobtrusive monitoring, bLL and uUL+uLL are reliable and practical to evaluate the amount and characteristics of everyday PA. For rehabilitative evaluation and planning, a combination of bUL and bLL is useful. To start with, bUL+bLL helps to evaluate the relationship between affected and unaffected limbs and activity limitations at early stage of stroke. When patient is able to transfer and stand, bLL is adequate to evaluate changes in PA and PAEE as rehabilitation progresses. Overall, bUL+bLL is the most accurate one to evaluate PA regardless of intensities of activities. However, clinicians and researchers should decide the model of accelerometers considering clinical settings and patient’s cooperation.

### Study limitations and strengths

This study has several limitations. First, currently there is no standard reference for MET multiple for people with disability [35]. 1 MET equivalent to 3.5 mLO_2_/kg/min is applied for the general population. Therefore, the Compendium of Physical Activities Tracking Guidelines was adopted to calculate PAEE measured by Actigraph and behavior mappings [36]. Second, the current study lacked heterogeneity in demographic characteristics due to a small sample size. Third, we could not evaluate reliability or validity of accelerometers for vigorous activities. Fourth, there is no valid algorithm for estimating energy expenditure in wrist or ankle accelerometers. Thus, the same algorithm used for hip accelerometers was applied to wrist and ankle accelerometers as previous studies did [37]. Fifth, we used PAEE (kcal/min) only for analysis to make results quantifiable and comparable to other objective methods including calorimetry and heart rate monitoring. Further studies need to use MET values to correct inter-individual differences in weight that could cause variations in kcal. Lastly, we were unable to test the data from accelerometers and behavior mappings against a standard method due to no definite gold standard to measure PA.

Nevertheless, our study has several strengths. This is the first study to prove the validity of accelerometers to monitor PA during diverse physical performances in a rehabilitative environment. Only one previous study has validated accelerometer in free-living environment and compared them to criterion method [38]. Only a couple of laboratory validations have been conducted in “simulated free living” conditions where participants repeat certain physical performances [11, 18]. Second, we used behavior mappings, a novel criterion method, to reinforce the validity of accelerometers by gathering details of PA including intensity, duration, types, and body postures. Third, we used a variety of combinations of accelerometers. This multi-sensor configuration helped us to detect fine activities from both upper and lower bodies, thus improving the categorization of PA intensities.

## Conclusions

In conclusion, 3-day accelerometeric recording is highly reliable for measuring daily PA of patients with stroke during inpatient rehabilitation. Overall, bUL+bLL is the most accurate one to evaluate PA. However, clinicians and researchers should decide the model of accelerometers considering clinical settings and patient’s cooperation: bUL or uUL for sedentary activities; bLL or uLL for light and moderate activities; bLL or uUL+uLL for unobtrusive everyday PA; a combination of bUL and bLL for rehabilitative evaluation and planning. Future studies involving patients with different stages of stroke and different severities of functional impairments are necessary.

## Supporting information

S1 Appendix List of activities based on MET reference from a Compendium of Physical Activities Tracking Guide expanded version. (DOCX)

S1 File The Anonymized data set. (XLSX)

S1 Text STROBE statement. (DOC)

## Abbreviations

AC: activity count
aLL: affected lower limb
aUL: affected upper limb
aUL+aLL: affected upper and lower limb
aUL+uLL: unaffected upper and lower limb
bLL: bilateral lower limbs
bUL: bilateral upper limbs
bUL+bLL: all limbs
EE: energy expenditure
ICC: intra-class correlation coefficient
MET: metabolic equivalent
PA: physical activity
PAEE: physical activity related energy expenditure
uLL: unaffected lower limb
uUL: unaffected upper limb
