# Supplementary material for: Feasibility, reliability, and validity of using accelerometers to measure physical activities of patients with stroke during inpatient rehabilitation
Source: PLoS One. 2018 Dec 31;13(12):e0209607. doi: 10.1371/journal.pone.0209607 (PMC6312264; doi:10.1371/journal.pone.0209607)
Supplement: S1 Appendix — (DOCX) [file pone.0209607.s001.docx]

**S1 Appendix. List of activities based on MET reference from a Compendium of Physical Activities Tracking Guide expanded version**

| **MET** | **Activity** |
| --- | --- |
| 0.9 | sleeping |
| 1 | lying quietly |
|  | reclining-writing, talking or talking on phone, reading, taking medication |
|  | sitting-doing nothing, watching television, listening to music (not talking or reading), on toilet, having hair or nails done by someone else |
| 1.2 | standing quietly |
| 1.3 | sitting-reading, book, newspaper |
| 1.5 | sitting-card playing or playing board games, talking or talking on the phone, arts and crafts with light effort, light office work, eating, bathing, meeting with talking or not |
| 1.8 | sitting -writing, desk work, typing, studying, general, including reading and/or writing |
|  | standing-talking or talking on the phone, reading, arts and crafts with light effort |
| 2 | sitting-arts and crafts with moderate effort |
|  | standing-getting ready for bed, talking and eating or eating only, showering, toweling off, household |
|  | standing or sitting- dressing or undressing, house hold, grooming(washing, shaving, brushing teeth, urinating, washing hands, putting on make-up) |
|  | walking or strolling very slow (less than 2.0mph) on level ground |
| 2.3 | walking very slow (less than 2.0mph) on level ground |
|  | mild stretching |
|  | hairstyling |
|  | standing-light work, talking, assembling |
| 2.5 | walking with slow pace (2.0mph) on firm surface |
| 2.8 | standing-playing with animals with light effort |
| 3 | stationary bicycling (50watts) with very light effort |
|  | standing-arts and crafts with moderate effort |
|  | walking downstairs |
| 3.5 | going up & down from floor |
| 5 | using crutch |
